# Clinical Interpretation and Management of Genetic Variants

**DOI:** 10.1016/j.jacbts.2020.05.013

**Published:** 2020-10-26

**Authors:** Ali J. Marian

**Affiliations:** Center for Cardiovascular Genetics, Institute of Molecular Medicine and Department of Medicine, University of Texas Health Sciences Center at Houston, Houston, Texas

**Keywords:** genetic testing, genetic variants, sequencing, CNV, copy number variants, HCM, hypertrophic cardiomyopathy, indel, insertion/deletion, LoF, loss of function, nsSNV, nonsynonymous single nucleotide variant, SNV, single nucleotide variant, SV, structural variant, WES, whole exome sequencing, WGS, whole genome sequencing

## Abstract

Genetic variants are major determinants of susceptibility to disease, response to therapy, and clinical outcomes. Advances in the short-read sequencing technologies, despite some shortcomings, have enabled identification of the vast majority of the genetic variants in each genome. The major challenge is in identifying the pathogenic variants in cardiovascular diseases. The yield of the genetic testing has been limited because of technological shortcomings and our incomplete understanding of the genetic basis of cardiovascular disorders. To advance the field, a shift to long-read sequencing platforms is necessary. In addition, to discern the pathogenic variants, genetic diseases should be considered as a continuum and the genetic variants as probabilistic factors with a gradient of effect sizes. Moreover, disease-specific physician-scientists with expertise in the clinical medicine and molecular genetics are best equipped to discern functional and clinical significance of the genetic variants. The changes would be expected to enhance clinical utilities of the genetic discoveries.

## Highlights

• The human genome contains approximately 4 million variants, whose population frequencies vary according to the ethnic backgrounds.
• Genetic diversity of humans in part determines interindividual variability in susceptibility to diseases, response to therapy, and the clinical outcomes.
• Genetic variants exert a gradient of biological and clinical effect sizes. In general, variants with the largest effect sizes are responsible for the single-gene disorders, whereas those with moderate and modest effect sizes are responsible for oligogenic and polygenic diseases, respectively.
• A phenotype is the consequence of nonlinear stochastic interactions among multiple genetic and nongenetic determinants.
• Discerning pathogenicity of the genetic variants, identified through genetic testing, in the clinical phenotype is challenging and requires complementary expertise in human molecular genetics and clinical medicine.

Genetic factors are important determinants of susceptibility to cardiovascular diseases, response to therapy, and the clinical outcomes. Whereas the contributions of genetic factors to cardiovascular diseases have been recognized for decades, the specific genetic variants that are pathogenic have remained largely undefined. Likewise, there is a paucity of knowledge on how to exploit the genetic information to prevent, attenuate, and treat the disease, at the population scale as well as at an individual level.

The foundation of the experimental genetics was laid by Gregor Mendel, who defined the fundamental principles of heredity in mid-19th century through analyzing crosses of the pea plants for selected traits. The elements of heredity, however, remained elusive to Mendel and others until the discovery of DNA by Friedrich Miescher a few years later and subsequent identification of purine and pyrimidine nucleotides by Albrecht Kossel at the beginning of the 20th century. These findings set the stage for identification of the elements of heredity through a set of complementary experiments. Oswald Avery and his colleagues elegantly showed that DNA, and not RNA, passes on the hereditary instruction in bacteria. The discovery was soon followed by Alfred Hershey and Martha Chase, who through a series of experiments in bacteriophage showed that DNA, and not the protein, was transmitted to the bacterial progeny. Consequently, DNA was established as the elements of heredity and provided an explanation for Mendel’s observations in the pea plant experiments.

Identification of DNA as the genetic material, about 75 years after the discovery of nucleic acids by Miescher, spearheaded a series of intense research to define the structure of DNA. Erwin Chargaff discovered that the number of purine (adenine and guanine) nucleotides equaled the number of pyrimidine (thymine and cytosine) nucleotides in the DNA. The finding coupled with the x-ray crystallographic images of DNA by Rosalind Franklin, guided James Watson and Francis Crick to describe DNA as a double-stranded helix formed by specific base pairings (1). The Watson-Crick specific base-pairs provided a copying mechanism for DNA replication.

The DNA replication machinery is extremely exquisite and precise. The major unit of this complex, namely the DNA polymerase, which was discovered by Arthur Kornberg in 1955, is blazingly fast and amazingly accurate, albeit it is not perfect. The replication machinery incorporates approximately 1 wrong nucleotide per every 100 million nucleotides that it synthesizes (the error rate is ∼1.3 × 10^−8^ per nucleotide) (2, 3, 4). Given the size of the human genome being approximately 3.2 × 10^9^ base pairs, the error rate of the DNA replication machinery introduces approximately 50 de novo point mutations and a lesser number of larger mutations with each genome replication (2, 3, 4, 5). Thus, each offspring differs from the parents by about 50 novel genetic variants. The replication error rate is not uniform across the human genome and varies according to complexity of the genome, with some spots being more prone to mutations (5, 6, 7). It is this rare error rate of the DNA replication machinery that is mainly responsible for human genetic diversity, and hence, the basis for variation in susceptibility to disease, response to therapy, and the clinical outcomes. The error, however, is not restricted to those occurring during DNA replication but also encompasses mutations that occur during recombination, DNA damage, and impaired repair mechanisms. For example, slippage strand mispairing, which typically occurs at the tandem repeats, leads to expansion of di- and trinucleotide repeats in the genome, which are the causes of the so-called triplet repeat syndromes (8). It is not unreasonable to surmise that the rare error rate of DNA replication is the essence of life, because in its absence, the eugenic human species would have been amenable to extinction by invading germs or diseases. The current pandemic of coronavirus-2019 (COVID-19), caused by severe acute respiratory syndrome-coronavirus-2 (SARS-CoV-2) virus, which is itself a product of a rare error of the replication machinery, is a prime example of the interindividual variability in susceptibility to COVID-2 and its clinical outcomes.

## Human Genetic Diversity

The discovery of the first restriction enzyme by Hamilton Smith and colleagues in 1970 enabled detection of differences among the individual genomes by the restriction fragment length polymorphism. However, the extent of the genetic variation among individuals was largely unrecognized until the development of dideoxynucleotide chain termination sequencing by Fred Sanger in 1977, which enabled sequencing at base pair resolution, and the advent of polymerase chain reaction by Kerry Mullis in 1985, which enabled amplifying the DNA fragments by more than a billion times. The advances led to the launching of the Human Genome Project in 1990, which aimed to sequence the entire human genome. The initial drafts of the human genome sequence, obtained from pooled genomes of several individuals, were successfully completed by The Human Genome Project and the Celera Genomics in 2001 (9,10). The completed sequence, albeit with some gaps, was published in 2003, and since then, has served as the reference genome (11). These initial genome sequencing, despite the incomplete coverage of the genome, catalogued over 2 million single nucleotide variants (SNVs), illustrating the vast genetic diversity of the humans.

The extent of genetic variations in an individual genome became further evident upon sequencing of the personal genomes of Craig Venter and James Watson, which illustrated the presence of about 4 million genetic variants, including a large number of small insertion/deletion (indel) and structural variants (SVs) in each genome (12,13). Collectively, sequencing of a large number of personal genomes have catalogued about 4 million SNVs, 100 thousand indels, and hundreds of SVs in each genome, which vary according to the ethnic backgrounds and geographic distributions of the populations, illustrating the genetic diversity of humans (Table 1) (14). Nevertheless, only a fraction of genetic variants in human genomes has been detected. The full extent of human genetic diversity is expected to be much greater than that observed so far.

**Table 1:** Approximate Number of Genetic Variants in the Genome of an Individual

Genetic variants	4 × 10^6^
SNVs	3.5 × 10^6^
Synonymous SNVs	11,000
Nonsynonymous SNVs	10,000
Small in-frame indel	130–178
Stop loss SNVs	4–14
Stop gain SNVs	67–100
Splice site disrupting SNVs	28–45
Small frameshift indels	192–280
Genes disrupted by large deletions	33–49
Total genes containing loss-of-function variants	240–345
SNVs designated as “damaging mutation”	48–82

indel = insertion/deletion; SNV **=** single nucleotide variant.

## Detection of the Genetic Variants

Clinicians are increasing challenged with interpretation of the results of genetic testing performed by third party providers as well as the failure in identifying the pathogenic variant(s) upon genetic testing. The lack of a commonly practiced approach to genetic testing further compounds the difficulty. Therefore, a brief discussion of the various steps involved in identification of the genetic variants using the current sequencing techniques is expected to inform physicians and the clinical investigators on the strengths and the shortcomings of the current approaches.

### Whole genome and exome sequencing techniques

Sequencing of individual genomes has become feasible with the development of new sequencing technologies, referred to as massively parallel sequencing or next generation sequencing. Technically, it is feasible to sequence the entire human genome, that is, whole genome sequencing (WGS), at a reasonable time and cost. Nevertheless, the cost of WGS increases as the coverage, that is, the number of sequence reads per each genomic fragment, increases and often becomes prohibitive for largescale sequencing. Because of the higher cost at a desirable coverage along with the computational requirements for analysis of the WGS output, the most commonly used approach focuses on sequencing genomic regions that code for proteins, namely, the exons. The approach of sequencing the exons, which encompass about 1% of the genome, is referred to as whole exome sequencing (WES). It is the commonly used approach for clinical genetic testing. The rationale for WES as opposed to WGS is based on the current data showing that the vast majority of the known disease-causing mutations, at least for diseases with Mendelian patterns of inheritance, affect the coding sequence. As indicated earlier, WGS adds considerable complexity to data analysis because of the extraordinary size of the sequence output. Nevertheless, with automation and advances in bioinformatics tools along with appreciation for variants in the noncoding regions, WGS is expected to become the preferred choice for detection of the genetic variants in each genome.

### Intrinsic shortcomings of the WGS and WES

Because clinical utilities of genetic testing heavily depend on the robustness of the detection methods, it is important to recognize strengths and weaknesses of the current and the evolving approaches. To begin, despite their names, neither WGS nor WES cover the whole genome or exome, because sequencing gaps exist in each approach due to various technical limitations. Consequently, detection of the variants in each genome is incomplete. In addition, WES and WGS are multistep processes, the key components of which include library preparation, sequencing, and variant detection. Each component has multiple steps that could introduce potential errors. WES and WGS require fragmentation of the genomic DNA (or RNA in the case of RNA sequencing) into approximately 50 to 100 base pair–long DNA fragments, and therefore, are referred to as short-read sequencing techniques. DNA fragmentation is followed by repairing the fragment ends, adding the adaptor for amplification by polymerase chain reaction, and library preparation. Whereas all DNA fragments are used to construct sequencing libraries in the WGS, an additional step is required for WES, which is capturing the DNA fragments that include the exons for construction of the sequencing library. Incomplete DNA fragmentation and poor library preparation could influence robustness of both approaches. In addition, the WES approach also has the inherent shortcoming of incomplete capture of all exons, and consequently, the risk of missing a fraction of the genetic variants that are located in the exons. Exome capture is influenced by a number of factors, including probe design, insert length in the library, uniformity of target regions, and a few other factors, which render complete capture of all exons at high depth challenging. With improved technology, the success rate of the current technologies for capturing the consensus coding sequence is >95%, albeit the consensus coding sequence does not adequately annotate all protein coding and, evidently, noncoding exons (15).

The short sequence reads are aligned, that is, mapped to the reference genome to identify the genetic variants. This is also an important step because inexact alignment could read to erroneous identification of the genetic variants. Approximately 80% to 90% of the reads are mapped to unique regions in the reference genome, making it feasible to identify the variants, assuming an adequate read depth. However, whenever the sequence reads are from the repeat regions in the genome or are from regions with a high level of sequence homology, proper alignment with the reference genome becomes challenging and increases the risk of missing or overcalling the variants in the repeat regions.

### Shortcomings of the reference genome

Once the short reads are aligned with the reference genome, nucleotides that differ from the reference sequences are identified as variants. As in the previous steps, confidence in calling a variant directly depends on the number of reads that cover that variant sequence. A low read number does not bode a high confidence in calling a variant. In addition, the reference genome to which the reads are compared has a number of shortcomings that influence accurate and comprehensive detection of the variants. The reference genome, which is a composite of a small number of genomes, does not adequately represent diversity of the human genomes. Consequently, a rare variant that is also present in the reference genome will not be called, and conversely, variants that are not present in the reference genome but are relatively common in another population will be called in the individual genome. This is particularly relevant to populations with different ethnic backgrounds, because variants are typically population-specific and naturally not expected to be adequately represented in the reference genome (16, 17, 18). Broadening the composition of the reference genome, although valuable in representation, is unlikely to be sufficient in assessing information content of the variants in the clinical setting. The reference genome also contains gaps, particularly in the complex genomic regions, which interfere with proper identification of the variants. Given that the reference genome is constructed on the basis of short-read sequencing, it does not adequately represent large indels or SVs, and hence, is not a robust reference point for their detection (19).

To overcome some of the shortcomings related to the reference genome, population-specific reference genomes comprising contiguous haploid sequence data of each chromosome are being generated (20). In addition, long-read single-molecule sequencing technologies are available that are capable of sequencing of several thousand bases of DNA and de novo assembly of each individual’s genome. The long-read sequencing approach is particularly relevant to sequencing of the repeat regions by increasing mapping certainty, as well as detection of variants located in the regulatory regions, and large SVs, which are increasingly being implicated in human diseases (reviewed in Eichler [21]). However, the error rate of current single-molecule sequencing in base-calling, and hence, accurate detection of SNVs and indels, is higher (3% to 15%) than that of short-read sequencing technologies, which limits clinical applications of these technologies (22). A number of approaches are being implemented to reduce the error rate and increase accuracy of variant calling by single-molecule sequencing technologies. Despite the relatively high error rate for small variant detection, the long-read single-molecule technologies are far superior to the short-read platforms for detection of the SVs (20). In view of the inherent limitations of each approach, a mixed approach, based on using various methodologies, ranging from long- and short-read sequencing techniques, array-based approaches, and chromosomal structural assays, is expected to become the preferred approach for discovering the genetic variants in each human genome.

The limitations discussed in the preceding text reduces the yield of WES, which is the most commonly used technique, in detecting the full spectrum of genetic variants in an individual exome in clinical genetic testing, which disappoints patients and physicians alike.

## Functional Spectrum of the Genetic Variants

The human genome contains a continuum of size variants that range from a single base to whole-chromosomal aneuploidies. The most common variants are SNVs, because each genome contains about 3.5 million SNVs (Table 1). Small indels are also common, but SVs are uncommon. However, SVs typically involve more nucleotides than SNVs because a fraction of SVs are large and encompass several million nucleotides (12,13). Ascertainment of functional significance of the genetic variants, in the absence of experimental data, is challenging and predictive. Various in silico algorithms have been developed, based on the known biochemical and biological properties of the involved genes and proteins, to predict functional significance of the variants. Experimental data, including studies in isogenic inducible pluripotent cell-derived cardiomyocytes, could provide valuable information for the functional significance of the genetic variants, albeit the findings are provisional to the experimental conditions, which are seldom physiological. Recently, genome editing has been successfully applied to garner functional data consistent with the clinical assessment of pathogenicity of a large number of SNVs in the *BRCA1* gene (23). Genome- or exome-wide application of the gene editing approaches to all possible or the candidate variants, once it becomes feasible, could facilitate functional characterization of the genetic variants.

A subset of the genetic variants is considered loss-of-function (LoF) variants, because they are expected to negatively affect function of the involved protein. The classic LoF variants include the nonsense or gain-of-stop codon variants, which lead to premature truncation of the protein. Likewise, canonical splice acceptor or splice donor variants are also expected to affect splicing of exons from introns, and hence, structure of the encoded protein. A small fraction of variants in each exome or genome lead to loss of the stop codon, and hence, result in elongation of the encoded protein. Functional significance of this subset of variants is variable, depending on characteristics of the involved protein and the elongated segment. A subgroup of nonsynonymous SNVs (nsSNVs) negatively affect function of the involved proteins and hence are also LoF variant (discussed in the following text).

### Nonsynonymous SNVs

They comprise a group of SNVs that affect amino acid sequence in the protein. NsSNVs are the main focus in clinical genetic testing, because the majority of the known mutations causing inherited disorders, such as cardiomyopathies or arrhythmogenic syndromes, are nsSNVs. Each genome contains about 11,000 to 13,000 nsSNVs, the vast majority of which are missense and a small fraction are nonsense mutations (Table 1). Distribution of nsSNVs is not random across the genome because some genes are more tolerant to LoF variants, whereas others show strong selection against them. For example, the *LMNA* gene, encoding lamin A/C, is intolerant to LoF variants, that is, LoF are very uncommon in the general population. By contrast, *TRIM63*, which codes for tripartite motif containing 63 (TRIM63), is very tolerant of LoF variants, that is, LoF variants are commonly found in this gene in the general population. This is an important feature that must be kept in mind when analyzing functional, and therefore, by inference, clinical significance of the nsSNVs.

A subset of nsSNVs are nonsense variants because such variants result in introduction of a premature stop codon and expression of aberrant transcripts. Transcripts containing premature termination codons are captured by the transcriptional surveillance and quality control mechanisms, and are targeted for degradation by nonsense-mediated decay. Likewise, whenever the variant eliminates a naturally occurring stop codon, that is, a loss of stop codon, the non-stop decay pathway identifies such variants and then subjects them to degradation. The net effect of these quality control measures is haploinsufficiency, which is a major mechanism in cardiovascular genetic disorders (24).

A number of structural- and conservational-based features that might affect stability of the protein structure are used to assess functional significance of the nsSNVs. The effects of the amino acid change on side chain polarity, charge, hydropathy, and helix, as well as topographic location of the involved residues on the protein (enzyme active site or catalytic domain), are important determinants of functionality of nsSNVs (reviewed in Tang and Thomas [25]). In addition to the intrinsic structural-based features of the involved amino acids, evolutionary conservation of the codon and the protein domain, as well as the population frequency of the variant, are important predictors of functionality. The final phenotypic effect of nsSNVs is a function of all of these features. There are approximately 2,500 nsSNVs at the conserved positions in the genome, of which only a small fraction has rare population frequency, typically defined at <1% (26). This subgroup is the most likely to be pathogenic. Most in silico prediction algorithms incorporate a combination of structural changes along with conservation state and population frequency to predict functional significance of a nsSNV, while giving different weights to various determinants of protein functionality.

### Synonymous variants

The genetic codon, comprising 3 nucleotides, is degenerate. The 4 nucleotides in the genome produce 64 different combinations of 3-nucleotide codons, of which 3 code for the stop codons and the remaining for the 20 amino acids. The number of codons for the amino acids vary from 1 for methionine and tryptophan to 6 for leucine and serine. The codon degeneracy leads to about 10,000 to 12,000 genetic variants in the coding regions that do not change the amino acid sequence in the protein and are referred to as synonymous or silent variants. By and large, synonymous variants are not expected to carry functional significance and are not known to cause diseases with Mendelian patterns of inheritance. However, synonymous SNVs could affect transcriptional and translational efficiency, splicing, and mRNA stability. A classic example of the functional and clinical significance of a synonymous variant is the c.1824C>T transition in exon 11 of the *LMNA* gene in patients with Hutchinson Gilford progeria syndrome (27,28). The variant does not affect the amino acid sequence (p.Gly608Gly), but instead activates a cryptic splice site and produces an LMNA protein without the last 50 amino acids at the carboxy terminus domain, called progerin. Likewise, synonymous SNVs could affect the tertiary structure of the RNA and the interaction of the RNA with microRNAs and other noncoding RNAs, exerting functional effects. Currently, there is no reliable predictor to assess functionality of the synonymous variants.

### Splice variants

The vast majority of the genes in the human genome comprise the regulatory regions, exons and introns. Only about 2.7% of genes in the human genomes do not have an intron and are referred to as intronless genes (29). Genes are transcribed as pre-mRNA or primary transcript, which contains both introns and the exons. The exon–intron boundaries are defined by splice consensus sequences, because introns typically start with GT and end with AG sequences, which are referred to as splice donor and acceptor sites, respectively. The motifs are recognized by the splicing machinery, comprising protein–RNA complexes, leading to excision of the introns and ligation of the consecutive exons. The process, along with 5′ capping and 3′ polyadenylation, leads to formation of the mature mRNA. Approximately 95% of the genes in the human genome have multiple splice variants (30). Genetic variants that are located at the consensus acceptor (5′) or donor (3′) splice sites could disrupt proper splicing of the primary transcript leading to exon skipping, retention of an intron, or a shift in the reading frame in the final mRNA product. Likewise, changes in the branch sequence, located 20 to 50 nucleotides upstream of the splice acceptor site, could induce alternative splicing (31). The SNVs could alter the existing canonical splicing sites, generate new sites, or activate a cryptic site (32). Splice variants typically results in in frame deletion of an exon and generation of a shorter protein. However, SNVs could also result in change in the coding frame and gain of new amino acids and loss of a significant domain, both of which could lead to premature truncation of the protein. Often transcripts containing a premature stop codon are degraded by nonsense-mediated decay, and hence, reduced expression level of the affected protein. A number of algorithms have been developed to predict the effects of genetic variants on splicing. Approximately, 10% of the all mutations that cause human genetic diseases affect the canonical splice sites. Peripheral blood transcripts, analyzed by RNA sequencing, could provide functional data on the effects of the variants on mRNA splicing, although splicing might be cell type-specific. Often, in vitro experiments are necessary to confirm biological effects of such variants.

### Regulatory variants

Functional significance of genetic variants located at the genomic regulatory regions, including those located at the promoter, enhancer, and 3′ regulatory regions, is difficult to determine, short of experimentation. SNVs located at the 5′ regulatory regions by changing binding affinity of the cognizant transcription factors could influence mRNA transcription. Likewise, those located at the enhancer regions could influence expression of multiple genes. Similarly, variants located in the 3′ untranslated regions have the potential to affect stability of the transcripts as well as binding of the mRNAs to microRNAs. In silico prediction of functionality of the SNVs located in the regulatory regions has not been reliable. Integration of genetic and transcriptomic data might provide valuable information in identifying functional regulatory variants while realizing that transcript levels are controlled at multiple levels.

### Intronic variants

All genes in the human genome with the exception of about 700 of approximately 20,000 genes contain introns (29). Introns comprise about 25% of the genome, whereas exons span <2%, and the remaining is the intergenic region (10). Genetic variants located in deep intronic (other than the canonical splice sites) and intergenic regions are the most abundant SNVs, totaling ∼4 million in each genome. Intronic variants are not expected to exert biological effects unless they generate new or cryptic splice sites, leading to inclusion of a pseudo-exon or a frame shift in the protein, affect the enhancers or the noncoding RNAs that are transcribed from that genomic regions.

### Insertion/deletions

Indels, which are generally defined as insertions or deletions affecting <50 nucleotides (definitions vary from 20 to 10,000 base pairs), are the second most common variants in the human genome. The number of indels in each genome vary from 100,000 to 1,000,000, depending the definition used (12,13,33). Short indels involving 1 to 3 nucleotides are the most common, comprising ∼70% of the indels in the genome (34). Likewise, deletion variants are twice as common as the insertion variants (34). Indels located in exons, that is, coding indels, comprise a minority of the indels, typically <1,000 in the genome. Coding indels typically result in deletion of 1 or multiple amino acids, that is, 3 nucleotides or multiples of 3 nucleotides, and maintain the open reading frame of the protein. Each exome contains about 85 and 25 coding indels that affect 3 and 6 nucleotides, respectively, and therefore maintain the coding frame (35). In-frame indels could also lead to a phenotype, as in the case of cystic fibrosis (36). About a third of the coding indels affect 1 or 2 nucleotides, corresponding to about 35 and 5 coding indels in each genome, respectively, and lead to a frame shift. Such coding indels often abolish expression of the involved protein and have considerable biological effects, depending on the tolerance of the gene to mutation. As for the noncoding indels, those involving 1 nucleotide are the most common, and their numbers correlate inversely with the number of affected nucleotides, that is, the smaller indels are more common than the larger indels (34). Indels located in the 5′ and 3′ regulatory regions could affect binding of the transcription factors to the regulatory elements necessary for transcription or instability of the mRNA, respectively. However, the majority of the indels, as the SNVs, are located in the intergenic regions, and prediction of their functionality is quite challenging.

### Structural variants

Large indels, typically more than 1 kilobase in size (by some definitions >50 base pairs), and genomic rearrangements are referred to as SVs. There is a diverse array of SVs, including deletion, insertion, inversion, translocation, variable number of tandem repeats, and tandem or dispersed duplication. Accurate identification of SVs in the genome has been difficult in part because of the shortcomings of short-read sequencing and the reference genome, as discussed earlier (reviewed in Eichler [21]). The existing data suggest the presence of several thousand SVs in each genome, which are nonrandomly distributed across the genome (37). The majority of the SVs are smaller than then 10 kilobases, whereas a few could encompass several million nucleotides; collectively, SVs involve more nucleotides than SNVs (38). Large SVs are rare in the population but are more likely to be functional (38). The majority of the SVs are copy number variants (CNVs), which comprise insertions and deletions, increasing or reducing the copy number of the affected genes, as opposed to inversions and other rearrangements. A number of CNVs encompass multiple genes and affect expression of the encompassed and surrounding genes, with those that increase the copy of the genes increasing their expression levels and vice versa. The association of CNVs with gene expression is to a large degree independent of SNVs and inadequately captured by the locus SNVs (39). SVs could affect gene expression through altering 3-dimensional remodeling of the chromatin, and consequently, their functional effects might be quite remote from the genomic locus wherein the SVs reside.

## Genetic Variants and Human Disease

The enormous genetic diversity of humans along with the phenotypic diversity of diseases have rendered genotype–phenotype correlation exceedingly difficult, if not impossible. The correlation inversely varies with the complexity of the phenotype. It is the worst for the complex phenotype and better, albeit far from perfect, for diseases with the Mendelian patterns of inheritance. Furthermore, correlation is the strongest for the most proximal phenotypes, such as effects of the genetic variants on the corresponding transcript levels; and weakest for the distance phenotype, which entails most clinical phenotypes, including mortality. Overall, genetic variants exert a gradient of effect sizes that partly determine the ensuing phenotypic effects, as discussed in the following text.

### Effect sizes of the genetic variants

Clinical significance of the genetic variants mirrors their functional/biological effects, albeit a higher threshold is required for the variants to impart a clinical effect than a functional effect. To elaborate, variants that impart large functional effects are likely to impart a clinically discernible phenotype. Conversely, those with no or a modest functional phenotype would not be expected to have a clinically discernible phenotype. Overall, it is fair to assume that biological and clinical effects of the genetic variants follow a gradient (Central Illustration). On 1 end of the spectrum are those variants that impart very large effect sizes and, therefore, are highly penetrant, which means those with the genetic variants exhibit the phenotype. These variants are typically responsible for the genetic diseases with Mendelian patterns of inheritance, such as hereditary cardiomyopathies and ion channel disorders. Clinical significance of such variants is evident in large families, wherein a near perfect cosegregation of the variant with the phenotype (genetic linkage) could be demonstrated. Variants with moderate-to-large effect sizes have incomplete penetrance in large families and are responsible for single-gene diseases detected in small families and sporadic cases. On the other end of the spectrum of the genetic gradient are variants that exert modest effect sizes, and these variants are responsible for the so-called complex phenotypes such as atherosclerosis and hypertension. Discerning clinical effects of the variants with modest functional effects is provisional to the function of the encoded protein and the involved biological pathways, as well as the complexity of the phenotype.

**Central Illustration:**
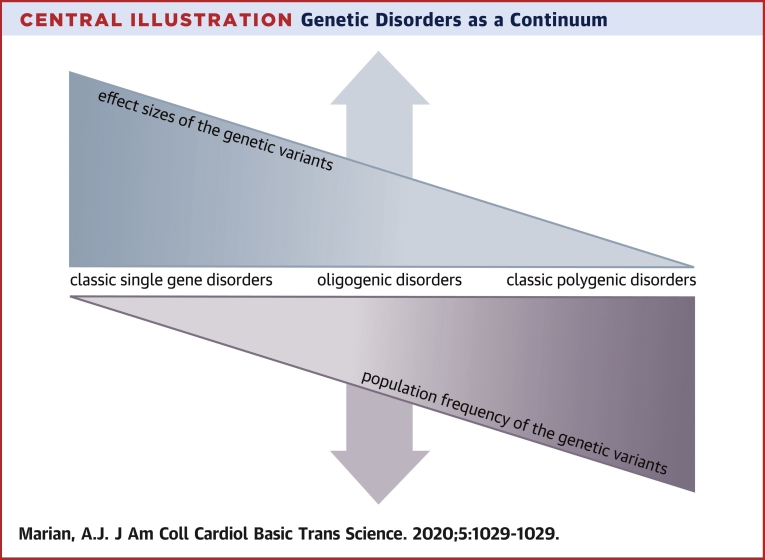
Genetic Disorders as a Continuum Genetic variants impart a gradient of functional and clinical effect sizes that vary from negligible to very large. Population frequency of the genetic variants inversely correlates with their effect sizes. Those with large effect sizes are rare and those with small effect sizes are common. The classic single-gene disorders result from the very large effects of rare variants, whereas the polygenic disorders result from the cumulative effects of multiple variants with small effect sizes. Most genetic disorders are in between the 2 ends of the spectrum.

Frequency spectra of the deleterious genetic variants and their effect sizes vary across the populations. A notable example are the founder mutations, which are detected in seemingly independent families that originate from common ancestors. As the population with the founder mutation drifts and migrates, the mutation aggregates within the population, although it remains exceedingly rare in the general population. The long QT syndrome in the European and South African populations is partly caused by founder mutations (40) (reviewed in Brink and Schwartz [41]).

### Pathogenic variants

Recognizing that the genetic variants impart a gradient of effect sizes, whether functional or clinical, the focus has been to identify those variants that impart clinically discernible effects, and hence, play a role in the pathogenesis of the disease. Such variants are referred to as the pathogenic variants. In the familial setting, the cause and effect relationship, that is, the pathogenic role of the genetic variants, could be ascertained by cosegregation analysis, as discussed earlier in the text. However, this is only possible when cosegregation could be analyzed in several family members for highly penetrant variants. It is inconclusive when the family size is small or the variants show low penetrance, that is, some family members carry the variant without showing the phenotype of interest. This challenge is further compounded by age-dependence of the penetrance, which means those with the candidate genetic variant might be free of the phenotype at the age when evaluated but develop the disease later in life. Evidently, cosegregation analysis is not feasible or informative in the sporadic cases or small families. In such cases, the focus is on identifying the pathogenic variants on the basis of their functional effects and population frequency, and inferring their causal role in the disease, as discussed in the following text. Finally, one has to consider the possibility that the candidate variant cosegregating with the phenotype might be in linkage disequilibrium with the actual pathogenic variant and not necessarily the true pathogenic variant.

The phenotype is the consequence of stochastic and nonlinear interactions among the genetic variants and the nongenetic factors (42,43). The genetic variant that imparts the largest effect size is the main pathogenic variant, which is often called the “causal” variant. The other variants, whenever pathogenic, are the modifiers of the phenotype (42, 43, 44, 45). The modifier variants lessen or accentuate the phenotype imparted by the causal variant, and their effects vary according to their interacting genetic networks (42). Consequently, the clinical phenotype associated with the pathogenic genetic variants, in particular its severity, that is, expressivity of the main pathogenic variant, is context-dependent because it is influenced by the modifier variants as well as nongenetic factors. The modifier variants also influence penetrance of the main pathogenic variant(s). The effects of the modifier variants on penetrance and expressivity collectively are in part responsible for variable phenotypic expression of the disease among family members. The modifier variants exert larger effects on the phenotype when the effect size of the causal variant is modest or moderate. Nevertheless, they also significantly influence penetrance of the causal variant with relatively large effect sizes (46).

Occasionally, there are multiple variants with large effect sizes, as in the case of digenic or oligogenic conditions. Nevertheless, the causal variant(s) is a single variant or comprises only a few, whereas the modifier variants are frequent. Consequently, the cumulative effect sizes of the modifier variants, particularly in the homozygous form, could be as large, if not larger than the effect sizes of the causal variant(s) (47,48). The notion further emphasizes focusing on the individual in assessing clinical significance of the genetic variants. Furthermore, it suggests the need for long-term longitudinal evaluation of families and the individuals in assessing pathogenicity of the genetic variants.

### Assessing pathogenicity of the genetic variants

Several guidelines have been advocated to assess pathogenicity of the genetic variants, as described elsewhere (49). Discerning pathogenicity of the genetic variants is challenging, let alone establishing the causality, which is almost impossible to unequivocally ascertain in a single individual. Given the gradient of effect sizes, dichotomous categorization of the variants into pathogenic and nonpathogenic is an oversimplification but is performed for practical reasons. Overall, genetic variants with predicted detrimental effects on gene/protein functions are likely to be pathogenic. Each human genome contains ∼300 LoF variants with potential clinical significance (Table 1) (17). Variants that lead to premature truncation of the encoded proteins, such as indels, stop gain, stop loss, and canonical splice site variants, are strong candidates to be pathogenic (Table 2). Likewise, variants that affect the initiation codon are expected to be pathogenic, because they are typically LoF variants. Such variants when heterozygous could annul expression of the affected allele, and if homozygous, expression of the gene in toto.

**Table 2:** Determinants of Pathogenicity

	Effects
Variants	
Start codon	May nullify expression of the affected copy of the gene
Nonsynonymous (missense) variants	The most difficult group to assess • De novo variants • Population frequency • Biochemical and biological effects, as discussed in the text • Cross-species conservation • Known pathogenic codon/hot spot • Effects on protein functional domain(s) • Relevance of the involved protein to the disease
Canonical splice sites	Those leading to frame shift are expected to deleterious as indels, but those resulting in alternating splicing or exon skipping might not impart clinical effects
Stop gain	Introduction of a premature stop codon and termination
Stop loss	Loss of the natural stop codon and elongation of the encoded protein with novel amino acids
3′ regulatory region	Could affect stability of the transcript
Indels	Frame shift indels are expected to lead to introduction of novel amino acids and premature truncation of the protein In-frame indels often are not pathogenic
SVs	Hard to detect with short-read sequencing, but large SVs are likely to be pathogenic
De novo variants	More likely to be pathogenic
Population frequency	Rare variants typically exert larger effect sizes than common variants
Tolerance of gene to LoF	Variants in genes intolerant to LoF are more likely to be pathogenic
Multiple transcripts	Involvement of the main transcript increases the chance of pathogenicity
Homologue of involved gene	Homologue might compensate
Tissue expression	Expression of the affected gene in the cell type affected by the phenotype
Biological pathway	Involvement of the affected genes in the biological pathways pertinent to pathogenesis of the phenotype

indel = insertion/deletion; LoF = loss-of-function; SV = structural variant.

The most challenging group of genetic variants, in terms of defining their clinical significance, is the missense variants, which are relatively abundant in each genome. Clinical significance of the missense variants in part mirrors their biological significance, as discussed earlier. In addition, the population frequency of the variant, topography of the variant on the involved protein, such as its location in an enzymatic activity domain, and the relevance of the protein to the pathogenesis of the disease are considered in determining pathogenicity. Moreover, identification of the same variant in multiple independent individuals with the phenotype of interest attests to pathogenicity of the variant. Finally, various resources, such as the NCBI database ClinVar and Genome Aggregation Database (gnomAD) are available and should be accessed to gather information in assessing pathogenicity of the genetic variants. Nevertheless, it is important to recognize that the group data might not be sufficiently robust to infer pathogenicity of a variant in an individual, because pathogenicity of a variant might be context-dependent, influenced by the modifier genetic variants as well as nongenetic factors.

In interpreting clinical significance of the functional variants, several additional factors have to be considered, including expression pattern of the gene, tolerance of the involved gene to LoF or gain of function variants, relevance of the involved gene to known pathways involved in the pathogenesis of the disease, and the presence of multiple mRNA transcripts as well as homologues of the involved genes (Table 2). As discussed earlier, population frequency of the variant is an important consideration, as rare variants are more likely to impart larger effect sizes than the common variants (17,50). Overall, the population frequency of the genetic variants inversely correlates with their effect sizes, because a common variant seldom exerts a large effect size. However, it is important to note that rare variants are population specific, and therefore, population frequency of the variants has to be assessed in that context of the specific population in which the variant is identified (16,18,26). In accord with the preceding, de novo variants, defined as variants detected in the index case, but absent in the parents, are more likely to be pathogenic. Each genome has about 50 to 60 de novo variants, the vast majority of which are not expected to exert an effect that is clinically relevant (Table 1). However, those located in genes pertinent to the pathogenesis of the disease of interest are strong candidates to be pathogenic, and hence, clinically significant.

The *TTN* gene, encoding the giant sarcomere protein titin, illustrates the challenges one faces in determining pathogenicity of the genetic variants. *TTN* is a well-established causal gene for dilated cardiomyopathy (51). Mutations in the *TTN* gene that are predicted to truncate the protein prematurely are considered responsible for about 15% to 20% of dilated cardiomyopathy cases (51, 52, 53). However, only a few *TTN* mutations have been identified that cosegregate with the phenotype, and hence, provide strong evidence of causality. The gene, which codes for about 35,000 amino acids, contains a large number of missense and truncating variants. It is tolerant to missense, but not truncating, variants. Whereas *TTN* missense variants are not expected to be pathogenic, the truncating variants are considered pathogenic (51). However, truncating variants are also found in the apparently healthy individuals in the general population, which renders the distinction between pathogenic and apparently benign truncating variants challenging. Applying the concept of the gradient of effect sizes, one may envision that *TTN* truncating variants impart a varying degree of functional effect, some of which induce an early clinical phenotype, some to a slowly developing phenotype later in life, and others to no phenotype at all. Consequently, to discern whether a truncating variant in the *TTN* gene is pathogenic, serial exquisite phenotypic characterization at the molecular and clinical levels would be necessary to detect the modest effect of some of the truncating variants in the *TTN* gene.

## Clinical Genetic Testing

Genetic variants are an important determinant of susceptibility to the disease, its clinical outcomes, and the response to therapy. Currently, clinical genetic testing is primarily used in families with single-gene disorders, such as hereditary cardiomyopathies and ion channel disorders, whereby identification of a pathogenic variant in the proband enables cascade screening of the family members who are at the risk of inheriting the variant, and hence, developing the disease. Cascade screening is probably the most clinically impactful application of genetic testing, because identification of those who carry the pathogenic variant will lead to early detection of the disease and possible interventions. Similarly, identification of the family members who have not inherited the pathogenic variant eliminate unnecessary serial clinical evaluation of such individuals, releasing the stress and burden of frequent clinical evaluation.

Genetic testing could lead to an accurate distinction of the disease from its phenocopy conditions. For example, over a dozen diseases, such as storage diseases, clinically mimic hypertrophic cardiomyopathy (HCM). In this scenario, genetic testing by leading to identification of the causal pathogenic variant would enable distinction of HCM from the phenotype conditions, such as Anderson-Fabry disease or glycogen storage diseases. The distinction is not inconsequential because the clinical course, as well as treatment of the disease and its phenocopy conditions, could vary. In the case of HCM and its phenocopy conditions, new specific therapies are being developed for HCM caused by mutations in genes encoding sarcomere proteins (54). Likewise, enzyme replacement therapy is indicated in Anderson-Fabry disease, a major HCM phenocopy (55). Similarly, RNA interference and small molecule targeting amyloidogenesis seem to be effective specific therapies for transthyretin amyloidosis (56,57).

The impact of genetic testing on clinical outcomes is less clear and is inconsistent across studies, although phenotypic correlation seems to less unstable when the causal gene rather than the specific mutation is considered (gene–phenotype rather than mutation–phenotype correlation). In general, the influence of genetic variants on distant phenotypes, such as clinical outcomes, is modest and confounded by a large number of genetic and nongenetic factors (58). Consequently, it is difficult to discern the modest effects of the genetic variants on distant phenotypes, including variants that are pathogenic for the disease. With the exception of a few studies, the findings of genotype–phenotype correlation studies are generally population-specific and have not been consistently reproduced across different studies (59).

Genetic testing might also enable individualization of therapy, as discussed earlier, in differentiating the phenocopy conditions from HCM, because for the former specific therapies might be available. Likewise, genetic testing in long QT syndromes might lead to specification of therapy, because treatment with a beta-blocker is highly effective in patients with long QT syndrome 1, caused by mutations in *KCNQ1*, but not as effective in those with long QT syndrome 3 due to pathogenic variants in the *SCN5A* gene (reviewed in Schwartz et al. [60]).

### Comprehensive versus focused genetic testing

The influence of the genetic variants on various aspects of cardiovascular medicine advocates for its wide-spread use, which has been made possible by the recent technical advances, despite the limitations of these techniques, as discussed earlier. The main impediment, however, is in discerning the clinical significance of the genetic variants identified through genetic testing, which was also discussed earlier. The current approach to genetic testing is focused on detection of the putatively pathogenic variants in genes implicated in the disease, including those associated with the phenotype, but their causal role is less certain. To avoid overinterpretation of the plethora of putative functional variants in the genome, some investigators have advocated limiting the genetic testing to genes that have a well-established causal role in the disease of interest, such as the genes coding for major sarcomere protein in HCM and those coding for major ion channels in arrhythmogenic syndromes (61, 62, 63, 64). The current approach, which is less restrictive and inclusive of various genes associated with the phenotype, regardless of strength of the evidence, has <50% yield for most cardiovascular diseases, including hereditary cardiomyopathies, which is disappointing to physicians and patients alike. Restriction of the genetic testing to only genes with robust evidence of causality is minimalistic, because it focuses only on the known genes, which account only for about one-half of the cases for most single-gene cardiovascular diseases. Therefore, the approach hinders discovering new causal genes for the genetic disorders and defining the full genetic spectrum of cardiovascular diseases. Likewise, it is based on the premise that the pathogenic variants impart very large effect sizes, that is, the low hanging fruit, which is only pertinent to a fraction of the cardiovascular disorders, further diminishing the yield of genetic testing. The approach is agnostic of the fact that genetic variants exert a gradient of effect sizes, and only a fraction of the pathogenic variants in single-gene disorders impart large effect sizes.

The conventional thinking has to be revisited to recognize the genetic disorders as a continuum and not dichotomous categories of single-gene and polygenic disorders. It is not surprising, but rather expected, that a subset of the so-called single-gene disorders, such as HCM, have oligogenic causes (65,66). Conversely, it is solidly established that a subset of complex traits, such as dyslipidemia or systemic arterial hypertension, are caused by mutations in a single gene or are oligogenic (67, 68, 69). Consequently, the boundaries of conventional categorization of genetic diseases are being refined to a continuum. To take advantage of this concept, comprehensive detection and analysis of all genetic variants, preferably through WGS, at least at the academic institutions, should be pursued in all patients with cardiovascular diseases. Otherwise, focusing on the known genes, along with the limitations of current approaches to genetic testing, will hinder the progress in our elucidating the genetic basis of cardiovascular diseases. Moreover, phenotypic characterization should be applied at the highest resolution possible to detect early and often subtle subclinical phenotypes in order to properly annotate pathogenicity of the variants in the clinical practice. The point was well-illustrated more than 2 decades ago upon detection of myocardial tissue Doppler abnormalities before development of overt HCM in individuals who carried pathogenic variants in genes encoding sarcomere proteins (70,71).

## Conclusions

Advances in the molecular genetic techniques have afforded the opportunity to sequence an individual genome, and hence, identify the large number of genetic variants that reside in each genome. Despite the remarkable advances, the approach is far from perfect and has significant shortcomings, which were discussed in the preceding text. These shortcomings are in part responsible for the relatively low yield of genetic testing in clinical practice. In addition, the restrictive approach to identification of the pathogenic variants, focused only on the major known genes, has further limited delineating the full spectrum of the genetic basis of cardiovascular diseases. To advance the field beyond the current, somewhat stagnant state, changes in 3 fronts are needed. First, it is necessary to overcome the current technological limitations of WES and WGS by shifting from the short-read sequencing approaches to long-read single-molecule sequencing platforms and incorporating complementary approaches. The shift will enable de novo assembly of each individual’s genome and will afford robust identification of the genetic variants. However, to implement long-read sequencing approaches in the clinical practice, the error rate of variant identification has to be reduced significantly. Second, the prevailing concept of considering the genetic variants as deterministic rather than probabilistic factors has to shift toward the latter, whereby the probability of the disease increases proportionately with the effect sizes of the genetic variants. Accordingly, the genetic disorders are a continuum rather than the dichotomous categories of single-gene or polygenic diseases. In this understanding, the classic monogenic and polygenic diseases are the opposite ends of the spectrum, encompassing the majority of the disorders, whose phenotypes are the consequence of stochastic and nonlinear context-dependent interactions among genetic variants, as well as nongenetic factors. Thirdly, comprehensive analysis of the clinical implications of the genetic variants would requires a level of scientific knowledge both in medicine as well as in genetics that is typically outside of the expertise of the clinical practitioner or the geneticist. Therefore, genetic exceptionalism must be abandoned, and genetics must be fully incorporated into cardiovascular medicine. A new breed of disease-specific physician-scientists who have garnered complementary expertise in clinical as well as genetic medicine should be trained. These unique individuals will fulfil the dictum of treating the patient who has the disease, as envisioned by Sir William Osler.

Given the focus on the present paper on clinical implications of genetic discoveries, it merits noting that the greatest impact of genetic variants is in delineating the fundamental mechanism(s) that govern the pathogenesis of the disease. The discovery of *PCSK9* as a causal gene for autosomal dominant hypercholesterolemia by Catherine Boileau’s group in 2003 typifies the true impact of genetic variants on the practice of medicine (68). The genetic discovery laid the foundation for the subsequent development of *PCSK9* inhibitors, which are highly effective in reducing plasma low-density lipoprotein cholesterol levels as well as reducing cardiovascular mortality (72,73). To conclude, the key is to discover the fundamental secrets of the nature and never to be concerned about the immediate clinical or translational impact of the discovery. The impact will become evident over time.

## Author Relationship With Industry

This work was supported in part by National Institutes of Health grant S10 OD018135, National Heart, Lung, and Blood Institute grants R01 HL151737, 1R01HL132401, and S10OD018135 Leducq Foundation grant 14 CVD 03, The Ewing Halsell Foundation, George and Mary Josephine Hamman Foundation, and the TexGen Fund from Greater Houston Community Foundation. Dr. Marian has reported that he has no relationships relevant to the contents of this paper to disclose.

## References

[1] Watson J.D., Crick F.H. (1953). Molecular structure of nucleic acids; a structure for deoxyribose nucleic acid. Nature.

[2] Kong A., Frigge M.L., Masson G. (2012). Rate of de novo mutations and the importance of father's age to disease risk. Nature.

[3] Palamara P.F., Francioli L.C., Wilton P.R. (2015). Leveraging distant relatedness to quantify human mutation and gene-conversion rates. Am J Hum Genet.

[4] Besenbacher S., Liu S., Izarzugaza J.M. (2015). Novel variation and de novo mutation rates in population-wide de novo assembled Danish trios. Nat Commun.

[5] Campbell C.D., Chong J.X., Malig M. (2012). Estimating the human mutation rate using autozygosity in a founder population. Nat Genet.

[6] Francioli L.C., Polak P.P., Koren A. (2015). Genome-wide patterns and properties of de novo mutations in humans. Nat Genet.

[7] Kloosterman W.P., Francioli L.C., Hormozdiari F. (2015). Characteristics of de novo structural changes in the human genome. Genome Res.

[8] Zhao X.N., Usdin K. (2015). The repeat expansion diseases: the dark side of DNA repair. DNA Repair (Amst).

[9] Lander E.S., Linton L.M., Birren B. (2001). Initial sequencing and analysis of the human genome. Nature.

[10] Venter J.C., Adams M.D., Myers E.W. (2001). The sequence of the human genome. Science.

[11] International Human Genome Sequencing Consortium (2004). Finishing the euchromatic sequence of the human genome. Nature.

[12] Levy S., Sutton G., Ng P.C. (2007). The diploid genome sequence of an individual human. PLoS Biol.

[13] Wheeler D.A., Srinivasan M., Egholm M. (2008). The complete genome of an individual by massively parallel DNA sequencing. Nature.

[14] Auton A., Brooks L.D., Durbin R.M., Genomes Project Consortium (2015). A global reference for human genetic variation. Nature.

[15] Parla J.S., Iossifov I., Grabill I., Spector M.S., Kramer M., McCombie W.R. (2011). A comparative analysis of exome capture. Genome Biol.

[16] Novembre J., Johnson T., Bryc K. (2008). Genes mirror geography within Europe. Nature.

[17] Abecasis G.R., Altshuler D., Auton A., Genomes Project Consortium (2010). A map of human genome variation from population-scale sequencing. Nature.

[18] Bergstrom A., McCarthy S.A., Hui R. (2020). Insights into human genetic variation and population history from 929 diverse genomes. Science.

[19] Chaisson M.J.P., Sanders A.D., Zhao X. (2019). Multi-platform discovery of haplotype-resolved structural variation in human genomes. Nat Commun.

[20] Chaisson M.J., Huddleston J., Dennis M.Y. (2015). Resolving the complexity of the human genome using single-molecule sequencing. Nature.

[21] Eichler E.E. (2019). Genetic variation, comparative genomics, and the diagnosis of disease. N Engl J Med.

[22] Ameur A., Kloosterman W.P., Hestand M.S. (2019). Single-molecule sequencing: towards clinical applications. Trends Biotechnol.

[23] Findlay G.M., Daza R.M., Martin B. (2018). Accurate classification of BRCA1 variants with saturation genome editing. Nature.

[24] Marston S., Copeland O., Jacques A. (2009). Evidence from human myectomy samples that MYBPC3 mutations cause hypertrophic cardiomyopathy through haploinsufficiency. Circ Res.

[25] Tang H., Thomas P.D. (2016). Tools for predicting the functional impact of nonsynonymous genetic variation. Genetics.

[26] Abecasis G.R., Auton A., Brooks L.D., Genomes Project Consortium (2012). An integrated map of genetic variation from 1,092 human genomes. Nature.

[27] De Sandre-Giovannoli A., Bernard R., Cau P. (2003). Lamin a truncation in Hutchinson-Gilford progeria. Science.

[28] Eriksson M., Brown W.T., Gordon L.B. (2003). Recurrent de novo point mutations in lamin A cause Hutchinson-Gilford progeria syndrome. Nature.

[29] Louhichi A., Fourati A., Rebai A. (2011). IGD: a resource for intronless genes in the human genome. Gene.

[30] Pan Q., Shai O., Lee L.J., Frey B.J., Blencowe B.J. (2008). Deep surveying of alternative splicing complexity in the human transcriptome by high-throughput sequencing. Nat Genet.

[31] Li M., Pritchard P.H. (2000). Characterization of the effects of mutations in the putative branchpoint sequence of intron 4 on the splicing within the human lecithin:cholesterol acyltransferase gene. J Biol Chem.

[32] Wimmer K., Roca X., Beiglbock H. (2007). Extensive in silico analysis of NF1 splicing defects uncovers determinants for splicing outcome upon 5' splice-site disruption. Hum Mutat.

[33] Wang J., Wang W., Li R. (2008). The diploid genome sequence of an Asian individual. Nature.

[34] Lin M., Whitmire S., Chen J., Farrel A., Shi X., Guo J.T. (2017). Effects of short indels on protein structure and function in human genomes. Sci Rep.

[35] Mullaney J.M., Mills R.E., Pittard W.S., Devine S.E. (2010). Small insertions and deletions (INDELs) in human genomes. Hum Mol Genet.

[36] Kerem B., Rommens J.M., Buchanan J.A. (1989). Identification of the cystic fibrosis gene: genetic analysis. Science.

[37] Lin Y.L., Gokcumen O. (2019). Fine-scale characterization of genomic structural variation in the human genome reveals adaptive and biomedically relevant hotspots. Genome Biol Evol.

[38] Sudmant P.H., Rausch T., Gardner E.J. (2015). An integrated map of structural variation in 2,504 human genomes. Nature.

[39] Stranger B.E., Forrest M.S., Dunning M. (2007). Relative impact of nucleotide and copy number variation on gene expression phenotypes. Science.

[40] Winbo A., Diamant U.B., Rydberg A., Persson J., Jensen S.M., Stattin E.L. (2011). Origin of the Swedish long QT syndrome Y111C/KCNQ1 founder mutation. Heart Rhythm.

[41] Brink P.A., Schwartz P.J. (2009). Of founder populations, long QT syndrome, and destiny. Heart Rhythm.

[42] Riordan J.D., Nadeau J.H. (2017). From peas to disease: modifier genes, network resilience, and the genetics of health. Am J Hum Genet.

[43] Marian A.J. (2002). Modifier genes for hypertrophic cardiomyopathy. Curr Opin Cardiol.

[44] Schwartz P.J., Crotti L., George A.L. (2018). Modifier genes for sudden cardiac death. Eur Heart J.

[45] Marian A.J. (2008). Genetic determinants of cardiac hypertrophy. Curr Opin Cardiol.

[46] Brink P.A., Crotti L., Corfield V. (2005). Phenotypic variability and unusual clinical severity of congenital long-QT syndrome in a founder population. Circulation.

[47] Daw E.W., Lu Y., Marian A.J., Shete S. (2008). Identifying modifier loci in existing genome scan data. Ann Hum Genet.

[48] Daw E.W., Chen S.N., Czernuszewicz G. (2007). Genome-wide mapping of modifier chromosomal loci for human hypertrophic cardiomyopathy. Hum Mol Genet.

[49] Richards S., Aziz N., Bale S. (2015). Standards and guidelines for the interpretation of sequence variants: a joint consensus recommendation of the American College of Medical Genetics and Genomics and the Association for Molecular Pathology. Genet Med.

[50] Altshuler D.M., Gibbs R.A., Peltonen L., International HapMap Consortium (2010). Integrating common and rare genetic variation in diverse human populations. Nature.

[51] Herman D.S., Lam L., Taylor M.R. (2012). Truncations of titin causing dilated cardiomyopathy. N Engl J Med.

[52] Roberts A.M., Ware J.S., Herman D.S. (2015). Integrated allelic, transcriptional, and phenomic dissection of the cardiac effects of titin truncations in health and disease. Sci Transl Med.

[53] Jansweijer J.A., Nieuwhof K., Russo F. (2017). Truncating titin mutations are associated with a mild and treatable form of dilated cardiomyopathy. Eur J Heart Fail.

[54] Heitner S.B., Jacoby D., Lester S.J. (2019). Mavacamten treatment for obstructive hypertrophic cardiomyopathy: a clinical trial. Ann Intern Med.

[55] Wilcox W.R., Banikazemi M., Guffon N. (2004). Long-term safety and efficacy of enzyme replacement therapy for Fabry disease. Am J Hum Genet.

[56] Maurer M.S., Schwartz J.H., Gundapaneni B. (2018). Tafamidis treatment for patients with transthyretin amyloid cardiomyopathy. N Engl J Med.

[57] Benson M.D., Waddington-Cruz M., Berk J.L. (2018). Inotersen treatment for patients with hereditary transthyretin amyloidosis. N Engl J Med.

[58] Marian A.J., Belmont J. (2011). Strategic approaches to unraveling genetic causes of cardiovascular diseases. Circ Res.

[59] Crotti L., Spazzolini C., Schwartz P.J. (2007). The common long-QT syndrome mutation KCNQ1/A341V causes unusually severe clinical manifestations in patients with different ethnic backgrounds: toward a mutation-specific risk stratification. Circulation.

[60] Schwartz P.J., Crotti L., Insolia R. (2012). Long-QT syndrome: from genetics to management. Circ Arrhythm Electrophysiol.

[61] Walsh R., Buchan R., Wilk A. (2017). Defining the genetic architecture of hypertrophic cardiomyopathy: re-evaluating the role of non-sarcomeric genes. Eur Heart J.

[62] Walsh R., Thomson K.L., Ware J.S. (2017). Reassessment of Mendelian gene pathogenicity using 7,855 cardiomyopathy cases and 60,706 reference samples. Genet Med.

[63] Thomson K.L., Ormondroyd E., Harper A.R. (2019). Analysis of 51 proposed hypertrophic cardiomyopathy genes from genome sequencing data in sarcomere negative cases has negligible diagnostic yield. Genet Med.

[64] Adler A., Novelli V., Amin A.S. (2020). An international, multicentered, evidence-based reappraisal of genes reported to cause congenital long QT syndrome. Circulation.

[65] Li L., Bainbridge M.N., Tan Y., Willerson J.T., Marian A.J. (2017). A potential oligogenic etiology of hypertrophic cardiomyopathy: a classic single-gene disorder. Circ Res.

[66] Gifford C.A., Ranade S.S., Samarakoon R. (2019). Oligogenic inheritance of a human heart disease involving a genetic modifier. Science.

[67] Brown M.S., Goldstein J.L. (1976). Analysis of a mutant strain of human fibroblasts with a defect in the internalization of receptor-bound low density lipoprotein. Cell.

[68] Abifadel M., Varret M., Rabes J.P. (2003). Mutations in PCSK9 cause autosomal dominant hypercholesterolemia. Nat Genet.

[69] Tada H., Kawashiri M.A., Nomura A. (2018). Oligogenic familial hypercholesterolemia, LDL cholesterol, and coronary artery disease. J Clin Lipidol.

[70] Nagueh S.F., McFalls J., Meyer D. (2003). Tissue Doppler imaging predicts the development of hypertrophic cardiomyopathy in subjects with subclinical disease. Circulation.

[71] Nagueh S.F., Bachinski L.L., Meyer D. (2001). Tissue Doppler imaging consistently detects myocardial abnormalities in patients with hypertrophic cardiomyopathy and provides a novel means for an early diagnosis before and independently of hypertrophy. Circulation.

[72] Ray K.K., Landmesser U., Leiter L.A. (2017). Inclisiran in patients at high cardiovascular risk with elevated LDL cholesterol. N Engl J Med.

[73] Schwartz G.G., Steg P.G., Szarek M. (2018). Alirocumab and cardiovascular outcomes after acute coronary syndrome. N Engl J Med.

